# Psychological Responses to Breast Density Notification: Empowerment or Anxiety?

**DOI:** 10.1111/hex.70704

**Published:** 2026-06-17

**Authors:** Ji Mou, Senzhong Zheng

**Affiliations:** ^1^ Taizhou First People's Hospital Taizhou Zhejiang China

Dear Editor,

We have carefully read the article entitled Psychosocial Impact of Breast Density Notification Through Breast Cancer Screening: A Qualitative Interview Study by Gram et al. Using semi‑structured interviews with 19 women who reported anxiety following breast density notification, this study explored the psychosocial impacts of such notifications and identified key themes, including empowerment, ambivalence, changes in risk perception, and increased vigilance. It provides valuable qualitative evidence for understanding the subjective experiences of receiving breast density notifications [1].

We particularly agree with the authors' perspective that breast density notification has dual effects: on the one hand, it may enhance women's health awareness and proactive engagement in their own care; on the other hand, it may trigger anxiety, worry, and elevated risk perception [1, 2]. This finding suggests that communicating breast density information is not merely a form of knowledge provision but profoundly shapes individuals' understanding of disease risk, screening outcomes, and subsequent behavioral decisions. Accordingly, this study carries practical implications for optimizing current policies on breast density notification [1].

Nevertheless, several aspects of this research warrant further discussion.

First, the study sample was restricted to women who explicitly reported feeling anxious due to their screening result letter in a prior survey. Thus, the sample reflects the experiences of those who have already developed an anxiety response, rather than representing all screening participants receiving breast density notifications. This limits the generalizability of the findings to some extent. In other words, while the paper effectively addresses how anxious women understand and experience such notifications, it is insufficient to characterize the overall psychological impact of breast density notification on the general screening population.

Second, the identification of anxiety relied primarily on a single item: Receiving the screening result letter made me feel anxious. As the authors noted, this item was not formally validated and only underwent face validity testing [1, 2]. Consequently, it likely captured an immediate subjective emotional response rather than a psychometrically robust, comparable state of anxiety in the clinical psychological sense.

Third, the study highlights increased vigilance, greater willingness to perform breast self‑examination, and heightened prioritization of screening as important behavioral intentions observed after notification. However, such self‑reported behavioral intentions do not equate to actual health benefits. As the authors acknowledged in their discussion, current evidence remains insufficient to confirm clear benefits from additional screening or self‑examinations [3, 4]. This increased vigilance may also be accompanied by excessive worry, medicalization, and the risk of unnecessary healthcare utilization [3, 5]. Further clarification is needed in future research to distinguish appropriate health vigilance from overreaction induced by risk information.

Overall, the study by Gram et al. offers an important contribution to understanding the psychosocial consequences of breast density notification. It also reminds us that the value of health communication lies not only in ensuring informed consent, but also in promoting accurate understanding, reducing misinterpretation, and avoiding unnecessary psychological distress [1, 2]. Future studies, including broader populations, employing robust psychological measurement tools, and incorporating real‑world behavioral outcomes, will help achieve a more comprehensive assessment of the actual benefits and harms of breast density notification.

Sincerely,

## Author contributions

All authors contributed to the study conception and design. Ji Mou performed the material preparation, and wrote the first draft of the manuscript. Senzhong Zheng reviewed and edited the manuscript. All authors read and approved the final version of the manuscript.

## Funding

The authors have nothing to report.

## Conflicts of Interest

The authors declare no conflicts of interest.

## Data Availability

The authors have nothing to report.
